# Accurate space-group prediction from composition

**DOI:** 10.1107/S1600576724004497

**Published:** 2024-06-18

**Authors:** Vishwesh Venkatraman, Patricia Almeida Carvalho

**Affiliations:** a Norwegian University of Science and Technology, 7491Trondheim, Norway; b SINTEF Materials Physics, 0373Oslo, Norway; c CEFEMA, Instituto Superior Tecnico, University of Lisbon, Lisbon, Portugal; Shiv Nadar Institution of Eminence, India

**Keywords:** space groups, random forests, machine learning, data sets, prediction

## Abstract

Predicting crystal symmetry simply from chemical composition has remained challenging. Several machine-learning approaches can be employed, but the predictive value of popular crystallographic databases is relatively modest due to the paucity of data and uneven distribution across the 230 space groups. In this work, virtually all crystallographic information available to science has been compiled and used to train and test multiple machine-learning models. Composition-driven random-forest classification relying on a large set of descriptors showed the best performance. The predictive models for crystal system, Bravais lattice, point group and space group of inorganic compounds are made publicly available as easy-to-use software downloadable from https://gitlab.com/vishsoft/cosy.

## Abstract 2

All available crystallographic information is used to train classification models for crystal systems, Bravais lattice, point groups and space groups. Reasonably accurate results can be achieved in space-group prediction if the classes used to train the models comprise more than 100 entries.

## 1. Introduction

Exhaustive screening via experimentation is prohibitive and identification of interesting compounds is increasingly reliant on efficient computational methods, such as high-throughput *ab initio* simulations (Hautier, 2019 ▸; Marzari *et al.*, 2021 ▸; Sun *et al.*, 2021 ▸) and machine learning (ML) (Axelrod *et al.*, 2022 ▸; Kusaba *et al.*, 2022 ▸; Venkatraman & Carvalho, 2022 ▸). Yet, establishing crystallographic information from first principles remains computationally expensive and requires an educated guess on candidate structures (Oganov *et al.*, 2019 ▸). On the other hand, ML methods demand large amounts of data and, although popular crystallographic databases have been used before, the predictions do not meet the expectations for practical use (Venkatraman & Carvalho, 2022 ▸).

Various strategies for structural representation have been adopted within ML, ranging from graph-based methodologies (Chen *et al.*, 2019 ▸) to atomic pair distribution functions (PDFs) (Liu *et al.*, 2019 ▸). In the latter approach, a convolutional neural network model achieved top-6 prediction accuracy of ≃90% for 45 heavily populated space groups (Liu *et al.*, 2019 ▸). Others have relied directly on powder X-ray diffraction patterns combined with data augmentation through simulation (Park *et al.*, 2017 ▸; Oviedo *et al.*, 2019 ▸; Suzuki *et al.*, 2020 ▸). In this context, the ensemble decision-tree model proposed by Suzuki *et al.* (2020 ▸) attained accuracies exceeding 90% for crystal system classification and surpassed 80% top-5 accuracy for space-group prediction. Structure-agnostic methodologies attempting to learn patterns/associations purely from com­pound composition have also been employed, often through deep learning (Liang *et al.*, 2020 ▸; Goodall & Lee, 2020 ▸; Kong *et al.*, 2021 ▸; Li *et al.*, 2021*a* ▸,*b* ▸). Against this background, Venkatraman & Carvalho (2022 ▸) have shown that, for sufficiently large and well represented databases, random-forest (RF) models using composition-based descriptors trained on the 50 most frequent space groups consistently result in better predictions than those obtained by deep learning, with 0.75–0.34 versus 0.65–0.25 F1 scores, respectively, depending on the particular crystallographic database employed to train the models.

All previous ML studies have used very limited data sets or focused on only around 50 heavily populated space groups due to paucity of data and class skewness of the existing crystallographic databases (Venkatraman & Carvalho, 2022 ▸; Liu *et al.*, 2019 ▸). In this work, we have augmented the data and enhanced class coverage by compiling virtually all crystallographic information available to science on inorganic compounds. Composition-driven models based on demonstrated ML approaches (Saal *et al.*, 2020 ▸; Alsaui *et al.*, 2022 ▸; Venkatraman & Carvalho, 2022 ▸) have been trained on this merged database (MERGED) and tested for predicting crystal system, cell centering, Bravais lattice, point group and space group. The models have been integrated into user-friendly public domain software to facilitate fast prediction of crystal symmetry.

## 2. Data sets

Data were compiled from three experimental databases, namely, the Crystallography Open Database (COD) (Vaitkus *et al.*, 2021 ▸), Pearson’s Crystal Data (PEARSON) (ASM International, 2021 ▸) and the Inorganic Crystal Structure Database (ICSD) (Zagorac *et al.*, 2019 ▸), and combined with data from two databases containing structures calculated with density functional theory (DFT), namely, Open Quantum Materials Database (OQMD) (Saal *et al.*, 2013 ▸) and Materials Project (MP) (Jain *et al.*, 2013 ▸).

For each of the primary data sets, the numbers in the chemical formulas have been normalized to 1 and rounded to the fourth decimal position. Compounds for which the formulas could not be parsed, as well as duplicate entries, were eliminated. Structures comprising only a single element or noble gas(es) were also excluded.

Polymorphism is exhibited by fewer than 8% of the compounds in experimental databases. Therefore, meaningful predictions for these multi-labeled entries are hindered by data scarcity, and thus compounds crystallizing in multiple space groups have been excluded from the experimental data sets. A comprehensive treatment of multi-labeled data in the context of polymorphism can be found elsewhere (Venkatraman & Carvalho, 2022 ▸).

Given the high proportion of poorly represented space groups across all repositories, experimental and theoretical databases were merged to augment the data. While stability conditions for compounds in experimental repositories are often not readily available, it can generally be assumed that compounds adopting a single structure have been solved under standard atmospheric conditions of temperature and pressure, rendering them stable or prevalent at such conditions. For theoretical databases, only entries with a stability indicator of *E*_hull_ = 0 were retained. The decision to exclude compounds with multiple stability domains from experimental repositories and those lying above the DFT convex hull from theoretical repositories enhanced the proportion of compounds stable at moderate temperatures. This approach established a common foundation for both types of data.

**Figure 1:**
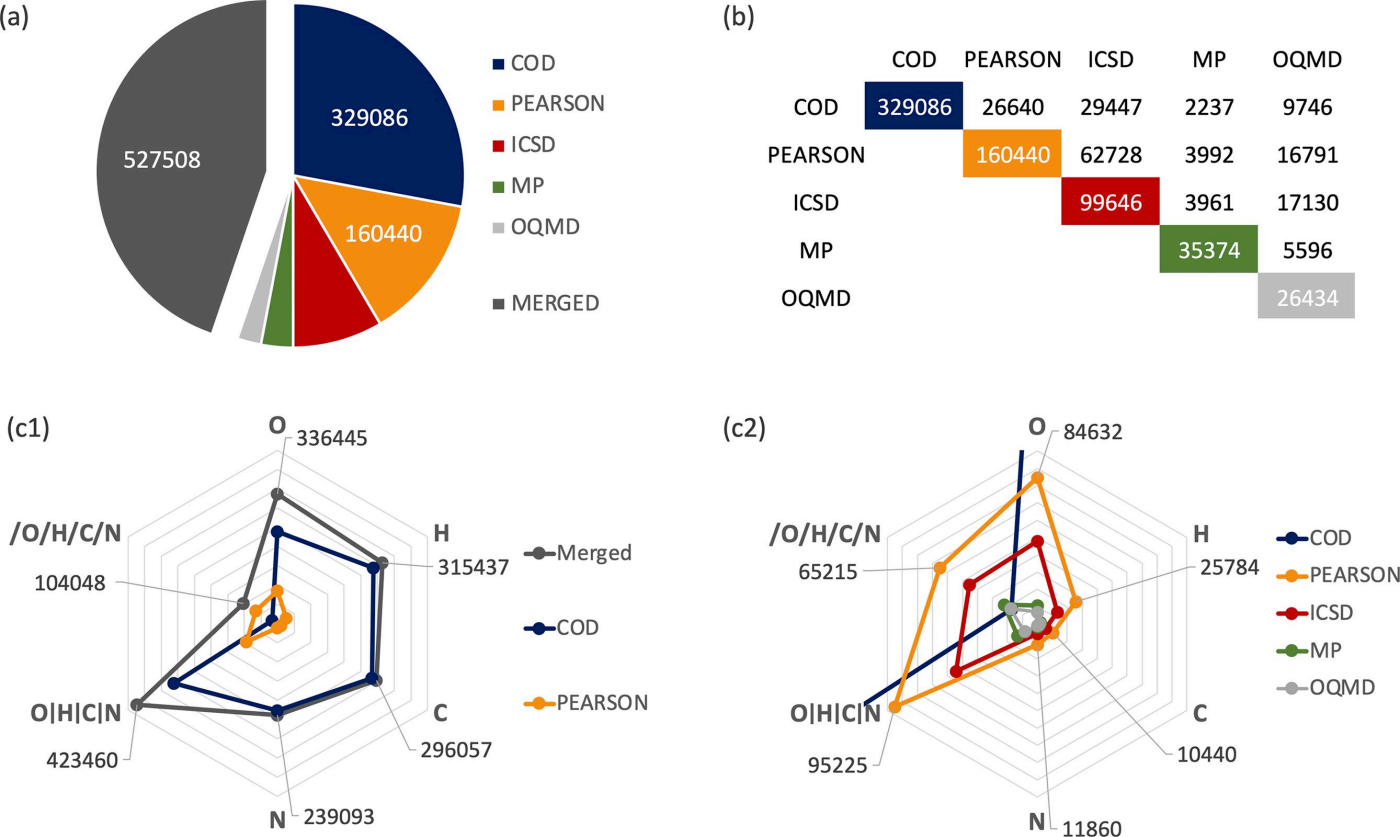
(*a*) Distribution of unique compounds in the databases. (*b*) Pairwise intersections. (*c*1) Element-containing compounds in each database, where O|H|C|N represents compounds containing at least one of these elements and /O/H/C/N represents compounds containing none of these elements. (*c*2) Magnified detail of (*c*1).

Fig. 1 ▸(*a*) represents the number of unique compounds in each database. After duplicate removal, the merged database comprised 527 508 unique compounds spanning 87 elements [see Table S1 in the supporting information (SI)]. Most data originate from the experimental databases, with the theoretical repositories offering less than 12% of the total number of compounds. Fig. 1 ▸(*b*) lists the pairwise intersections between the different data sets. Significant overlap exists between the experimental commercial data sets (PEARSON contains 63% of ICSD and ICSD contains 39% of PEARSON) and between these and OQMD (ICSD contains 65% of OQMD and PEARSON contains 64% of OQMD).

Actinium and polonium are the least common of the 87 elements present in MERGED (respectively, 0.06 and 0.006% of compounds). On the other hand, most compounds contain light elements such as oxygen (64%), hydrogen (60%), carbon (56%) and nitrogen (45%). Their distribution across the different databases is shown in Figs. 1 ▸(*c*1) and 1 ▸(*c*2). Com­pounds with at least one of the frequent light elements, *i.e.* O|H|C|N, account for 95% of the entries in COD, 59% in PEARSON, 55% in ICSD, 37% in MP and 32% in OQMD which, after duplicate removal, yielded 80% in MERGED. This proportion, significantly lower than that in COD, reflects the contribution to diversity imparted by the smaller databases with higher fractions of compounds without the frequent light elements [/O/H/C/N, see Figs. 1 ▸(*c*1) and 1 ▸(*c*2)]. Indeed, the large COD repository comprises a high fraction of mineral structures, which typically contain light elements, whereas the other primary databases seem more application oriented.

**Figure 2:**
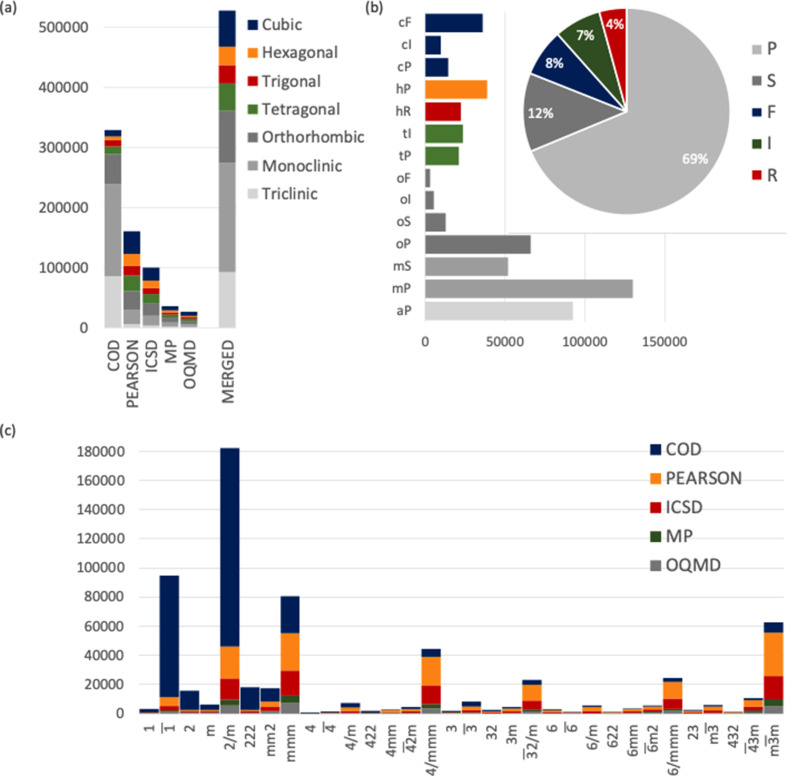
(*a*) Compound distribution across the crystal systems in all databases. (*b*) Distribution of compounds across the Bravais lattices and lattice centering types in MERGED. (*c*) Contribution of each primary database to each point-group class.

Fig. 2 ▸ breaks down the compound distribution across (*a*) the seven crystal systems for all databases, (*b*) the 14 Bravais lattices and five lattice centering types for MERGED, and (*c*) the 32 point groups for the primary databases. The monoclinic crystal system is the dominant class in MERGED, followed by the orthorhombic and triclinic systems. The predominance of these systems is essentially inherited from COD [see Fig. 2 ▸(*a*)]. The other primary databases show more balanced crystal system distributions and therefore contribute to enhancing the tetragonal, trigonal, hexagonal and cubic classes in MERGED [see Fig. 2 ▸(*a*)]. The dominant centering type in MERGED is *P* due to the high number of compounds with triclinic, monoclinic and orthorhombic primitive lattices, while the remaining centering types show more balanced proportions [see Fig. 2 ▸(*b*)]. In the primary databases each crystal system exhibits a clearly preponderant point group: 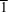 for triclinic, 2/*m* for monoclinic, *mmm* for orthorhombic, 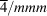 for tetragonal, 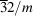 for trigonal, 6/*mmm* for hexagonal and 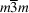 for cubic [see Fig. 2 ▸(*c*); a similar overall point-group distribution was obtained for MERGED (as shown in Fig. F1 of the SI), *i.e.* duplicate removal did not change the preponderant point groups in each crystal system].

**Figure 3:**
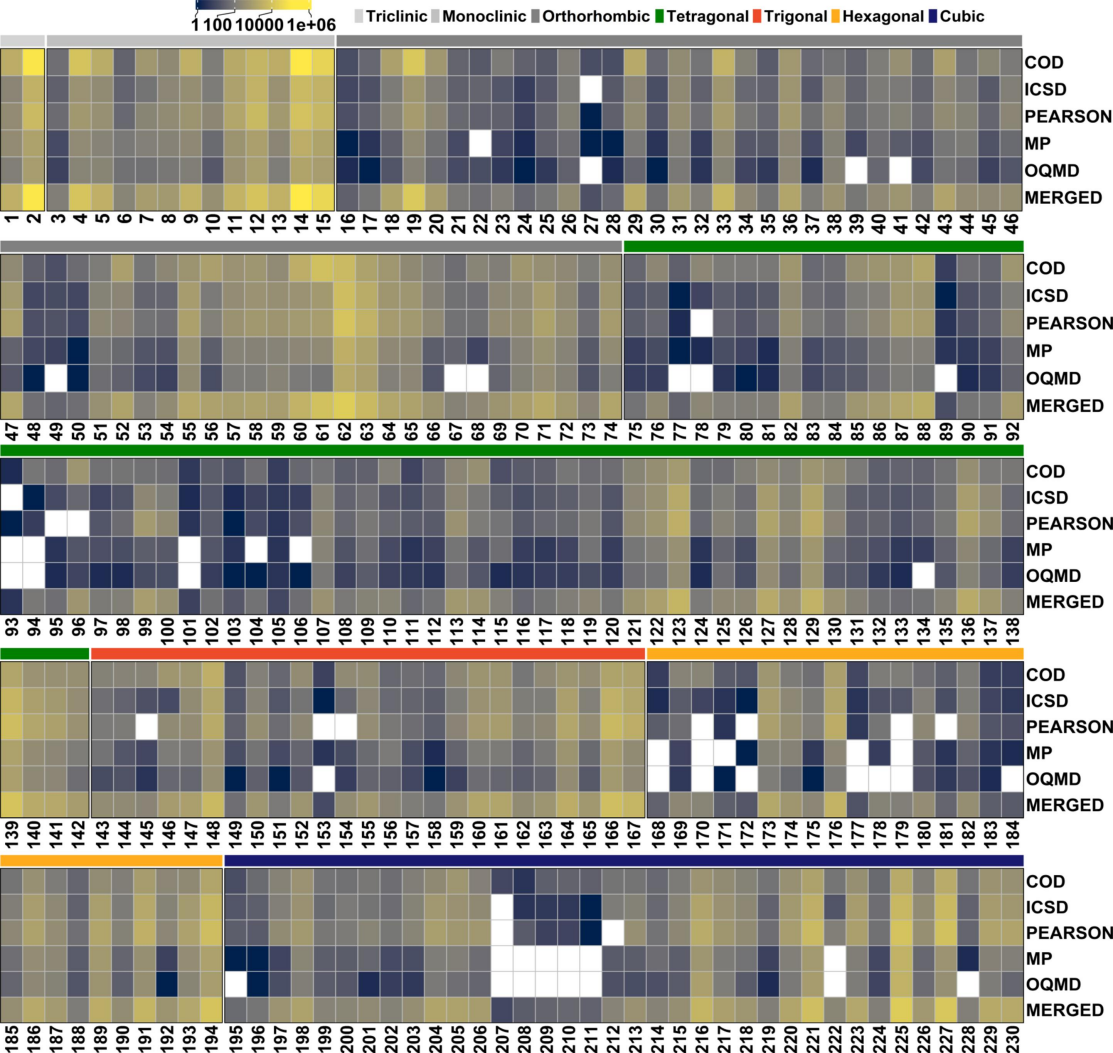
Distribution of unique compounds across the 230 space groups in the data sets.

**Figure 4:**
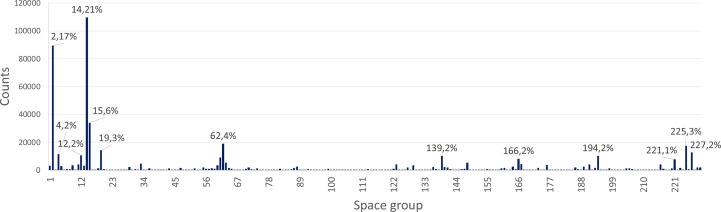
Space-group distribution in MERGED. The labels over frequent classes indicate the space-group number followed by the corresponding percentage. The number of compounds for each space group is listed in Table S2 in the SI.

The heatmap in Fig. 3 ▸ reveals important differences between the primary databases in terms of space-group representation. MERGED shows an overall improved coverage, with nearly 75% of the space groups comprising more than 100 compounds. Nonetheless, five space groups, namely, 93, 101, 105, 153 and 207, still contain fewer than ten compounds in the merged database. Meanwhile, space group 14 is the most populated, with over 100 000 compounds originating mostly from COD, but with significant contributions from PEARSON and ICSD. Despite the variations, all primary databases show highly frequent 2, 14, 15, 62, 225 and 227 space groups (see Fig. 3 ▸). The histogram in Fig. 4 ▸ further illustrates the uneven class occupation in MERGED. The striking preference of crystallization for specific space groups, leading to extremely frequent versus rare space groups, is a well known fact but a complex problem in crystallography and, to the best of the authors’ knowledge, clear space-group filling rules are yet to be defined for inorganic compounds [see the review by Urusov & Nadezhina (2009 ▸)].

## 3. Modeling

In a previous study (Venkatraman & Carvalho, 2022 ▸), the predictive performance of an ensemble decision-tree RF approach was compared against a deep-learning framework named Roost (Representation Learning from Stoichiometry), reported by Goodall & Lee (2020 ▸) to outperform even ElemNet, an alternative neural network method (Jha *et al.*, 2018 ▸). For each of the primary databases, the RF approach showed an overall better performance in multi-class classification (Venkatraman & Carvalho, 2022 ▸). Notably, Roost yielded disappointing results except for OQMD, which is severely skewed towards cubic structures, fostering cubic predictions and thereby justifying the earlier reports [only OQMD was employed by Goodall & Lee (2020 ▸) to test Roost and by Jha *et al.* (2018 ▸) to test ElemNet]. Here, for the augmented data set MERGED, we expand the palette of tools and analyze the prediction performance of

(i) the ensemble decision-tree RF approach relying on a large set of composition-based descriptors, which has been effectively tested in previous studies (Venkatraman, 2021 ▸, 2023 ▸; Venkatraman & Carvalho, 2022 ▸),

against two other ML approaches:

(ii) DUET, which combines bagging with boosting decision-tree methods via two classifiers (Vargaftik & Ben-Itzhak, 2022 ▸). A bagging model (RF-based) is trained on the entire training data set and, subsequently, a boosting model (XGBoost) is trained on a fraction of the data set for which the bagging model underperformed. To rank how valuable a given labeled sample is to training the boosting classifier, a heuristic called ‘data instance predictability’ is used to define the data set fraction for training the boosting model. The predictability-driven fraction of the training data set was set to the recommended value of 6% (Vargaftik & Ben-Itzhak, 2022 ▸).

(iii) TabNet, a deep-learning architecture for tabular data (such as a matrix of descriptor vectors) that uses sequential attention to select features from which to reason at each decision step (Arik & Pfister, 2019 ▸).

For each of the three approaches employed, the compounds were split into calibration (80%) and test (20%) sets. For each compound, a descriptor vector based on maximum, minimum, fraction-weighted mean and mode, as well as average deviations of the elemental properties (such as electronegativity, atomic weight, polarizability and number of filled/unfilled valence orbitals), was calculated using software written in Java (available from https://github.com/vvishwesh/MaterialDescriptors). The descriptor set includes other variables derived from element properties, such as specific heat and atomic packing efficiency (Guo *et al.*, 2011 ▸), as well as different electronegativity scales (Rahm *et al.*, 2019 ▸), not included in the original Magpie set (Ward *et al.*, 2016 ▸). The descriptor vector contained missing values due to the non-availability of complete sets of elemental attributes. Thus, a cleaning step was applied where descriptors with missing values were removed. This was followed by a correlation-based variable reduction step to exclude highly correlated variables (a pairwise squared correlation cutoff of 0.90 was used), which resulted in data sets with 126–129 descriptors (the variation stems from the random selection of the train/test sets). Finally, a fivefold cross-validation was carried out to assess the generalizability of the models (potential performance on unseen data). This was repeated three times with independent train/test splits to examine performance variability.

Space-group classes with less than 100 compounds have been excluded from the evaluation. The number of classes was hence seven for crystal systems, five for lattice centering, 14 for Bravais lattices, 32 for point groups and 172 for space groups (since in MERGED still 58 of the 230 space groups comprised less than 100 compounds). The assessment of the models was based on top-*k* accuracy, *i.e.* on the proportion of compounds for which a correct answer is present in the top-*k* results. Here, we have evaluated the accuracy for *k* = 1, 2, 3, 5.

## 4. Results and discussion

### 4.1. Cross-validation

**Figure 5:**
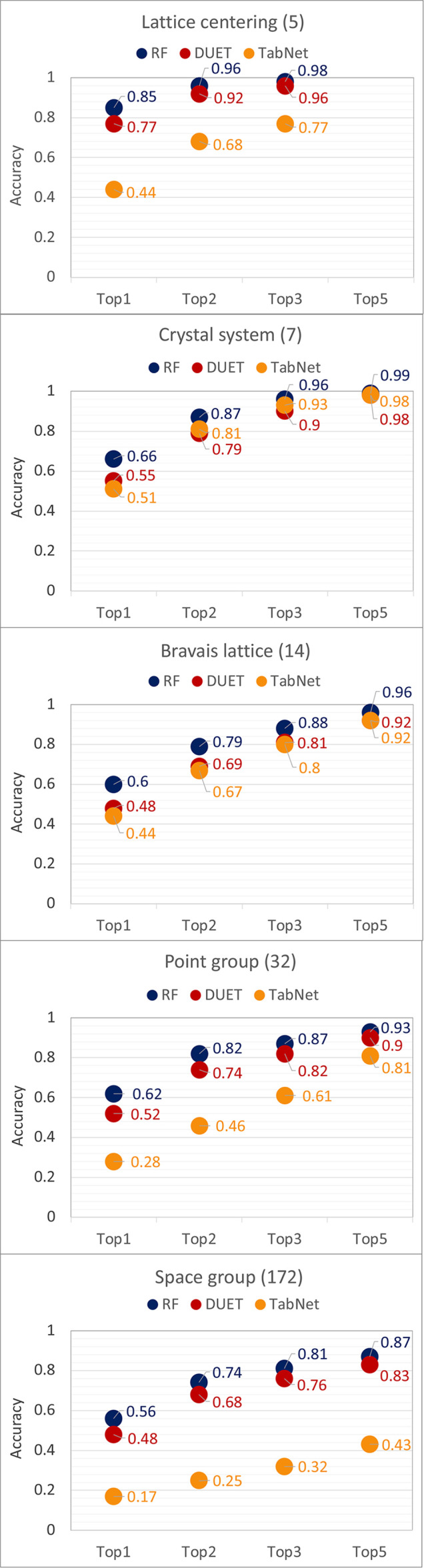
Top-*k* accuracies for the test set (averaged over three independent splits) obtained by the different ML approaches. Values in brackets indicate the number of classes associated with each response.

Fig. 5 ▸ provides a visual summary of the models’ performance in predicting lattice centering, crystal system, Bravais lattice, point group and space group for the test sets. As expected, the overall prediction quality decreases with the number of classes (in brackets). The RF model achieved the highest top-*k* accuracies, followed by DUET. The accuracies for TabNet were considerably lower, except for crystal system prediction where the values are marginally close to those of the other approaches. The performance gaps are more significant for space-group prediction, with top-5 accuracy of only 0.43 ± 0.03 for TabNet. In contrast, RF achieved top-3 accuracy of 0.81 ± 0.001 and above in all symmetry classifications (see labels in Fig. 5 ▸; the standard deviations for each response can be found in Table S4 in the SI). Baseline performance (wherein no predictors were used and instead the target values were averaged in some way) was assessed using the *basemodels* package in R through dummy classifiers on the following basis: (i) the most frequent class in the training set was selected for all instances, and (ii) class labels were assigned according to the class distribution in the training set. The multi-class Cohen’s kappa (Artstein & Poesio, 2008 ▸) values for these two dummy models across all training data sets were found to be close to 0. In comparison, the RF models exhibited values in the 0.5 to 0.6 range, confirming their robustness and generalization capability.

**Figure 6:**
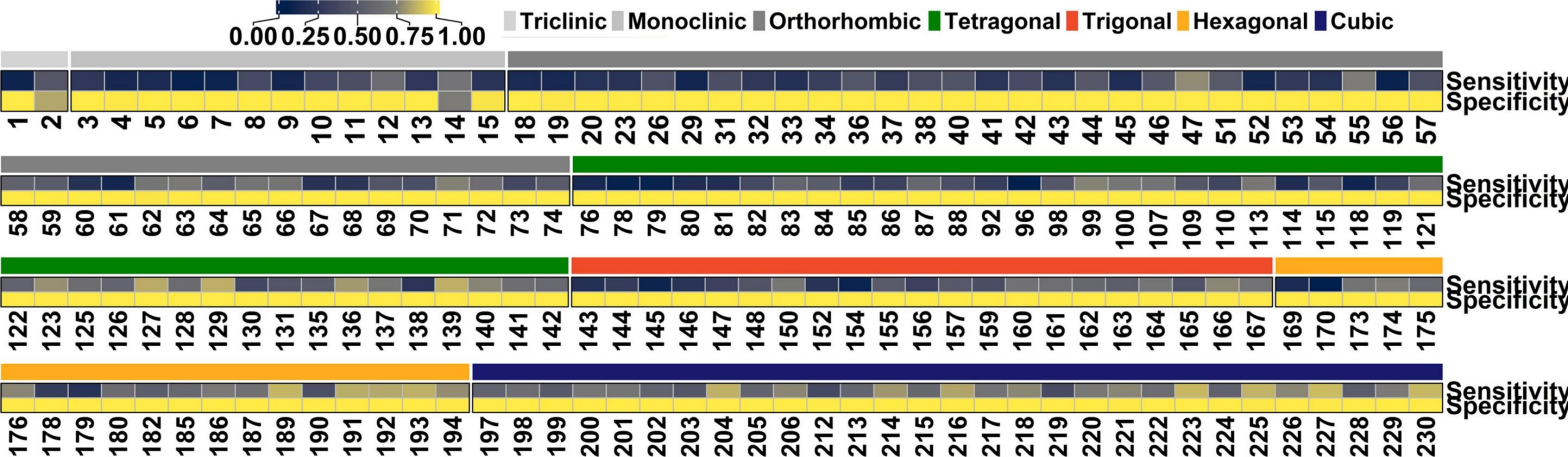
Heatmap showing the per-class sensitivity and specificity of the RF model for the 172 space-group test set.

In order to understand the reasons behind the relatively low top-1 accuracy for space-group prediction, we examined the class-wise performance of the RF model. The per-class sensitivity and specificity variations (Fig. 6 ▸) show that the model typically has high specificity but low sensitivity. This variability in performance is seen, in particular, for the monoclinic (space-group numbers 3–9), orthorhombic (18–23, 29, 52, 56, 60, 61) and tetragonal (76–82, 96, 118, 119) crystal systems. The poor discrimination power can be attributed to the paucity of data for some space groups (less than 200 compounds for space groups 3, 6 and 32, for instance). Class imbalance is also an issue for accuracy which, as an evaluation metric, is more meaningful when the class labels are uniformly distributed.

Overall, both the TabNet and DUET models yielded poorer predictive performance than the RF-based models. For the TabNet models in particular, studies by Kadra *et al.* (2021 ▸), Shwartz-Ziv & Armon (2022 ▸) and Grinsztajn *et al.* (2022 ▸) have shown that methods such as TabNet or other deep tabular data modeling approaches do not outperform popular ensemble approaches such as XGBoost and other tree-based models. Importantly, Kadra *et al.* report that, for almost 40 different types of tabular data sets, decision-tree models were seen to perform strongly even against specialized neural architectures.

### 4.2. External validation

While the RF models exhibit high accuracy when predicting the symmetry of test sets, the performance for completely unseen data may provide more realistic estimates. To this end, we compiled two independent data sets: (i) compounds extracted from the *American Mineralogist* crystal structure database (AMCSD) (Downs & Hall-Wallace, 2003 ▸) and (ii) data on high-entropy alloys and compounds (HEAC) collated from multiple sources (see Table S3 in the SI). The validation data sets were prepared as described for the primary databases.

AMCSD comprises 8253 compounds not present in MERGED that could be employed for external validation. However, since 85 of them belong to space groups outside the 172 classes on which the model was trained, only 8168 compounds (distributed across 160 space groups) have been used for validation of space-group prediction. Nevertheless, the entire disjoint set has been considered for the other symmetry categories. The HEAC set is limited to 125 novel compounds restricted to 15 space groups (albeit highly skewed towards 194 and 225, see Table S3 in the SI), all of which are included in the 172 classes used to train the space-group model.

**Figure 7:**
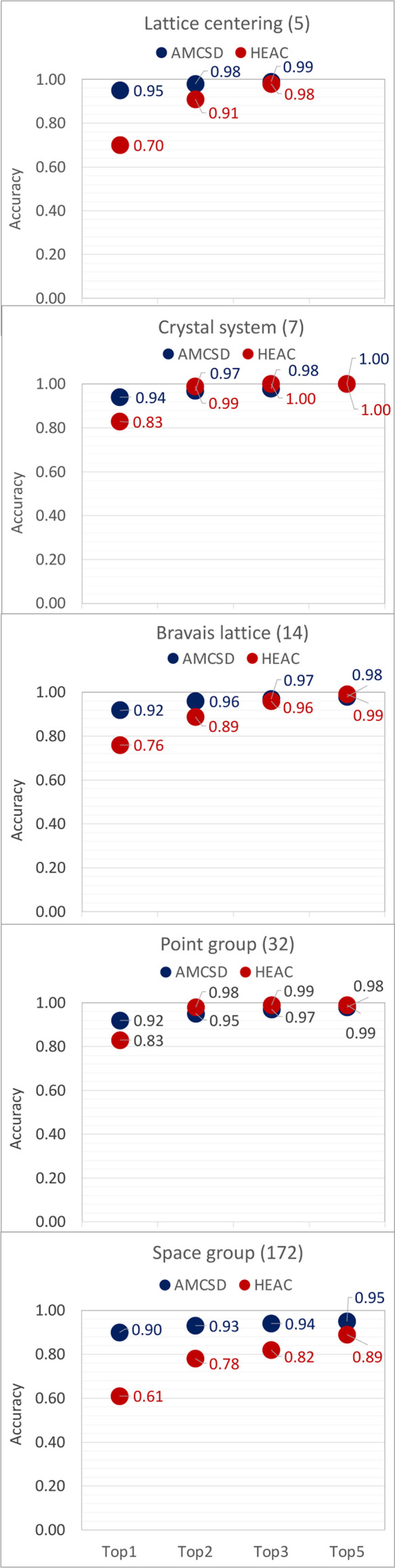
Top-*k* accuracies of the RF-based models for two independent test sets: (i) the AMCSD (Downs & Hall-Wallace, 2003 ▸) data set containing 8253 compounds and (ii) the HEAC data set comprising 125 compounds.

The top-*k* accuracies achieved in predicting the symmetry of the external data sets are presented in Fig. 7 ▸. For lattice centering, crystal system, Bravais lattice and point group, the predictions are highly accurate. Yet, the top-1 and top-2 performances are typically better for AMCSD than for HEAC. The space-group model yielded excellent metrics for AMCSD, with top-*k* accuracies exceeding 0.9 for all outcomes, while consistently lower performance was obtained for HEAC. Rather than only *per se* or against each other, the results achieved with external validations should also be evaluated in terms of the performance attained with the test sets (compare Fig. 7 ▸ with Fig. 5 ▸). In this scenario, several aspects are worth consideration:

(i) In the HEAC data set, each entry consists of five or more elements. The results achieved suggest that this characteristic is sufficiently well represented in the models. Indeed, MERGED comprises a relatively high fraction of compounds with more than four elements (55%, see Fig. F2 in the SI).

(ii) The AMCSD and HEAC data sets are comprised of, respectively, 10 and 83% of compounds without the prevalent light elements, while MERGED contains 20% of the /O/H/C/N compounds (see Fig. 1 ▸). The external validation shows that the amount of training data allowed the models to capture the crystallization behavior of both extremes, although this factor is likely to have contributed to the poorer prediction of the HEAC symmetries.

(iii) The external validation was carried out by training the models on all the data in MERGED, while only 80% was employed for internal validation. However, as demonstrated by the small standard deviations obtained for the three test splits (see Table S4 in the SI), the improvements with the training set augmentation are expected to be residual.

(iv) The AMCSD data set comprises more cubic and hexagonal and fewer triclinic compounds than MERGED, while the other crystal systems show similar distributions (see Fig. F5 in the SI). The HEAC data set consists essentially of high-symmetry compounds (89% cubic and hexagonal, see Table S3 in the SI). The better performance achieved with external data than with the test sets, particularly for HEAC, suggests that the models may better recognize the descriptor patterns associated with high symmetry.

**Figure 8:**
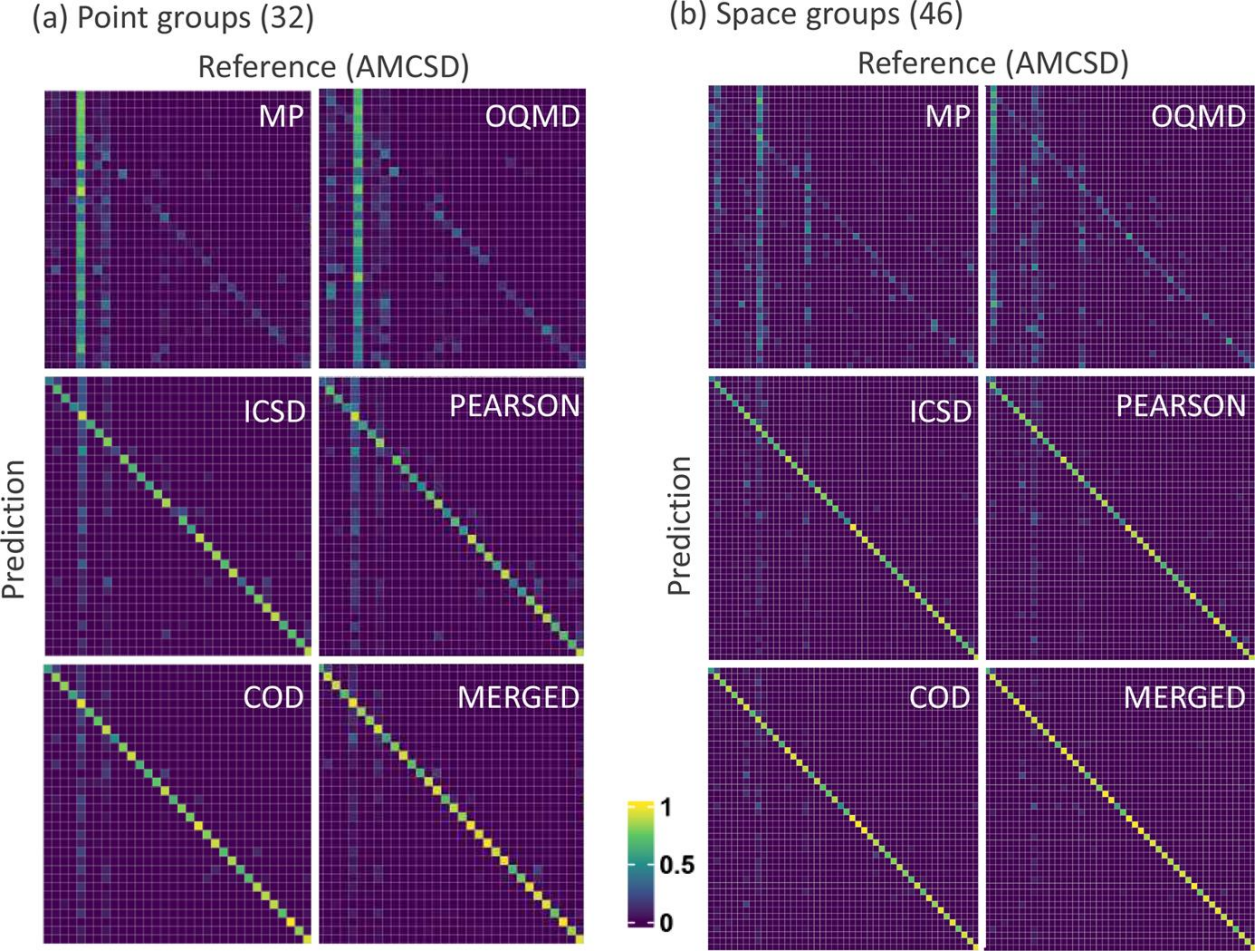
Confusion matrices for symmetry prediction using RF-based models trained on all databases (top-1 accuracy). (*a*) Point groups of the compounds in AMCSD. (*b*) Space groups of the compounds in AMCSD (for visualization clarity the RF-based models were trained only on the top 46 space groups of each database). Additional details can be found in Figs. F3 and F4 in the SI.

(v) The very high accuracies obtained for AMCSD, particularly for space-group classification, indicate that the external compounds may be stoichiometrically similar to those present in MERGED. The effects of data augmentation on AMCSD prediction can clearly be appreciated by comparing the confusion matrices (top-1 accuracy) when training the models on the different primary databases and on MERGED (see Fig. 8 ▸). The performance of models trained on COD and MERGED is high and similar as expected for mineral data. The stoichiometry similarity was further tested by changing the decimal position when rounding the numbers in the chemical formulas during the data preparation step. Rounding to the third decimal position reduced the AMCSD compounds not present in MERGED to 4374, while rounding to the second decimal position reduced the number of dissimilar compounds to 1320. The decimal position of rounding is however not a trivial matter since, for critical elements, precision is required to define the crystal structure adopted. Clearly, much work remains to be done in crystallographic data curation.

In summary, this work shows that high accuracy in symmetry prediction can be achieved by a decision-tree-based approach using solely elemental composition and the crystallographic information already available to science. In fact, the quality of the composition-driven prediction is notably higher than that obtained with models based on X-ray diffraction data (Suzuki *et al.*, 2020 ▸; Aguiar *et al.*, 2020 ▸; Corriero *et al.*, 2023 ▸) and atomic PDFs (Liu *et al.*, 2019 ▸), and also higher than for other descriptor-based ML approaches (Liang *et al.*, 2020 ▸; Zhao *et al.*, 2020 ▸; Li *et al.*, 2021*a* ▸). In the context of polymorphism, the predicted symmetry is expected to correspond to polymorph(s) (meta)stable at atmospheric conditions, since these are the standard circumstances for the entries in crystallographic databases.

### 4.3. Variable importance

**Figure 9:**
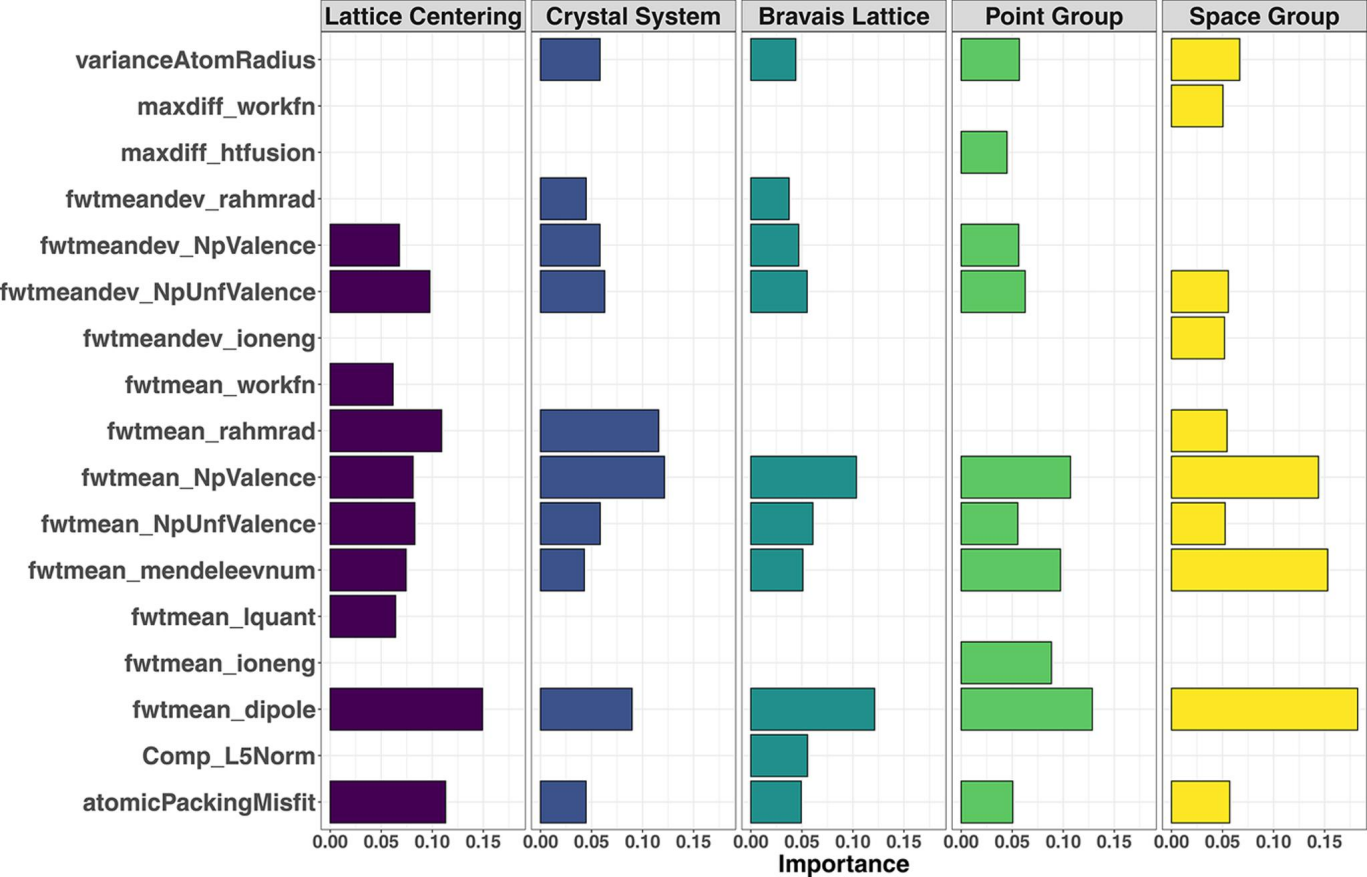
Variable importance in the RF models for lattice centering, crystal system, Bravais lattice, point group and space group. For brevity, only the ten most influential variables are shown for each symmetry category. The length of the bars is a quantitative measure of the decrease in accuracy upon removal of the variable from the set of descriptors. Gaps, *i.e.* absence, of a bar for a variable in a symmetry category indicate that the variable is not among the top-ten contributors. More information on each descriptor can be found in the SI.

The influence of each variable was evaluated from the decrease in accuracy upon its removal from the descriptor set. Fig. 9 ▸ shows the variable importance for the five different symmetry categories (for brevity only the top-ten variables are shown). Several top-ranking variables are shared between the symmetry categories, albeit with varying impacts on the response as revealed by the different lengths of the bars, while some are specific to the symmetry category. Important descriptors include the fwtmean_dipole and the fwtmeandev_NpUnfValence that point to the weighted mean values of the atomic dipole and unfilled *p* orbitals, respectively. The atomicPackingMisfit is an indicator of the atomic packing efficiency (Guo *et al.*, 2011 ▸; Wang *et al.*, 2015 ▸). Another variable with a dominant influence on the model outcome is the Mendeleev number (mendeleevnum), which can be seen as a combination of important properties such as atomic size and electronegativity into a single parameter (Allahyari & Oganov, 2020 ▸). This information establishes the background for a fundamental definition of the filling rules for space groups in inorganic compounds.

## 5. Concluding remarks

The present work demonstrates that an ensemble decision-tree-based approach can achieve high accuracy in the symmetry prediction of new compounds using solely their elemental composition and the crystallographic information already available to science. Although class skewness is an intrinsic property of crystallographic databases, we have shown that reasonably accurate results can be achieved in space-group prediction if the classes used to train the models comprise more than 100 entries. Currently, the best ML approaches are limited to about 172 space groups with sufficient data out of the 230 classes. Therefore, specific efforts to populate the sparse classes must be made to fulfill a sound information goal and accelerate the discovery of materials with unusual space groups. Another critical aspect is the stoichiometric precision in chemical formulas, which requires suitable curation so that unique compounds can be accurately discriminated. On a final note, a meaningful contribution of ML to crystallography in the context of polymorphism will require the availability of significantly more data on polymorph structures, as well as suitably curated stability ranges in terms of temperature and pressure.

## 6. Related literature

The following references are cited in the SI: Chellali *et al.* (2019 ▸), Chen *et al.* (2020 ▸), Fu *et al.* (2021 ▸), Gao *et al.* (2018 ▸), Generalic (2020 ▸), Gild *et al.* (2016 ▸, 2019 ▸), Glawe *et al.* (2016 ▸), Gould & Bučko (2016 ▸), Guedri *et al.* (2021 ▸), Jadhav *et al.* (2021 ▸), Joseph *et al.* (2020 ▸), Jung *et al.* (2021 ▸), Lilensten *et al.* (2014 ▸), Liu *et al.* (2020 ▸, 2021*a* ▸,*b* ▸), KnowledgeDoor (2020 ▸), Manglam & Kar (2022 ▸), Marik *et al.* (2019 ▸), Mayandi *et al.* (2021 ▸, 2022 ▸), Motla *et al.* (2022 ▸), Nygard *et al.* (2020 ▸), Oses *et al.* (2020 ▸), Rahm *et al.* (2019 ▸), Rost *et al.* (2015 ▸), Sharma *et al.* (2018 ▸), Tekgül *et al.* (2022 ▸), Uporov *et al.* (2020 ▸), Witte *et al.* (2019 ▸), Wu *et al.* (2021 ▸, 2022 ▸), Yusenko *et al.* (2017 ▸), Zhu *et al.* (2020 ▸), Zlotea *et al.* (2019 ▸).

## 7. Data and software availability

The data sets used in this study are available from the corresponding public repositories – OQMD (https://oqmd.org), Materials Project (https://materialsproject.org), COD (https://www.crystallography.net/cod). The ICSD and Pearson databases require commercial licenses. The prediction models have been added to an easy-to-use graphical user interface for public use. Instructions for software download and usage can be viewed at https://gitlab.com/vishsoft/cozy.

## Supplementary Material

Supporting tables, figures and descriptor calculation. DOI: 10.1107/S1600576724004497/ui5003sup1.pdf

Sample scripts for downloading OQMD and MP databases. DOI: 10.1107/S1600576724004497/ui5003sup2.zip
