# Supplementary material for: Accurate space-group prediction from composition
Source: J Appl Crystallogr. 2024 Jun 18;57(Pt 4):975–85. doi: 10.1107/S1600576724004497 (PMC11299606; doi:10.1107/S1600576724004497)
Supplement: Supplementary file 1 [file j-57-00975-sup1.pdf]

JOURNAL OF
APPLIED
CRYSTALLOGRAPHY

Volume 57 (2024)

Supporting information for article:

Accurate space-group prediction from composition

Vishwesh Venkatraman and Patricia Almeida Carvalho

**Element**	**Count**	**Element**	**Count**
Ac	304	Na	25539
Ag	14370	Nb	10818
Al	31844	Nd	10010
Am	209	Ni	31961
As	9960	Np	823
Au	8935	O	336445
B	40265	Os	3312
Ba	20855	P	78316
Be	2050	Pa	330
Bi	11069	Pb	10369
Br	22970	Pd	14814
C	296057	Pm	497
Ca	22444	Po	32
Cd	12054	Pr	7646
Ce	10733	Pt	12136
Cl	85249	Pu	870
Cm	97	Rb	6811
Co	32201	Re	6059
Cr	13216	Rh	9671
Cs	8838	Ru	16327
Cu	45770	S	79287
Dy	7598	Sb	12581
Er	6165	Sc	4882
Eu	8186	Se	15894
F	66067	Si	46602
Fe	51969	Sm	7831
Ga	13815	Sn	14109
Gd	9278	Sr	19171
Ge	15186	Ta	6307
H	315437	Tb	6845
Hf	3704	Tc	762
Hg	4699	Te	9261
Ho	5092	Th	2169
I	15835	Ti	21321
In	9868	Tl	4774
Ir	9071	Tm	3428
K	22008	U	5289
La	18160	V	12675
Li	18066	W	12464
Lu	3903	Y	12683
Mg	19006	Yb	5906
Mn	30353	Zn	25640
Mo	16319	Zr	12204
N	239083		

Table S1: Element-wise distribution in the merged dataset.

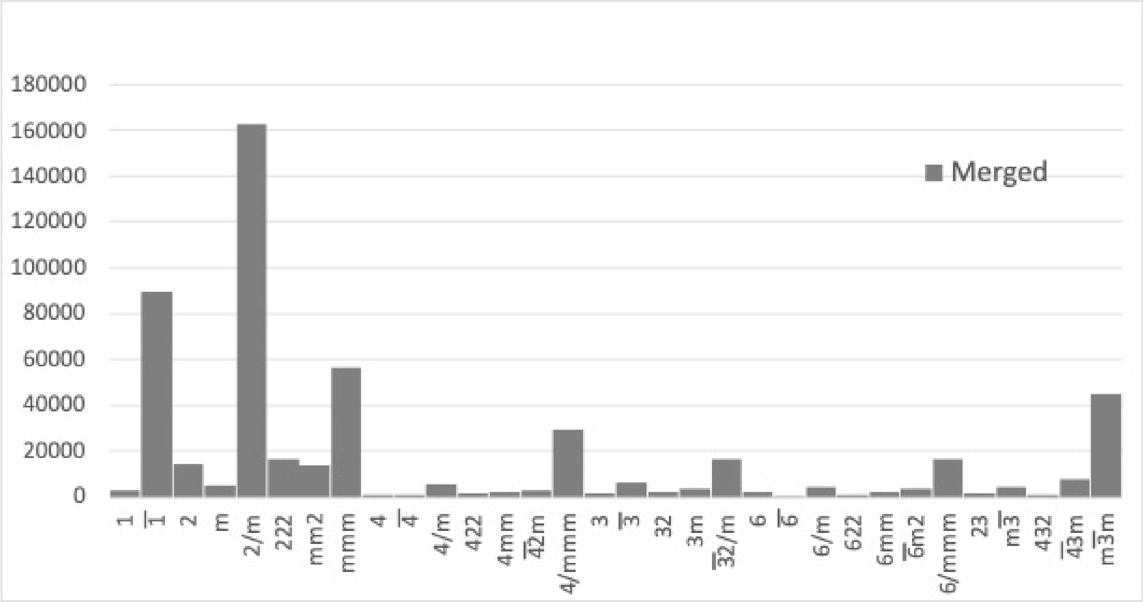

Figure F1: Point group distribution the the merged database.

SG	Count	SG	Count	SG	Count	SG	Count	SG	Count
SG001	3120	SG047	1633	SG093	3	SG139	10467	SG185	326
SG002	89466	SG048	52	SG094	79	SG140	2207	SG186	2003
SG003	114	SG049	34	SG095	52	SG141	1856	SG187	722
SG004	11485	SG050	51	SG096	544	SG142	837	SG188	97
SG005	2964	SG051	369	SG097	86	SG143	287	SG189	2524
SG006	167	SG052	1331	SG098	124	SG144	253	SG190	282
SG007	836	SG053	132	SG099	887	SG145	174	SG191	4269
SG008	702	SG054	263	SG100	367	SG146	785	SG192	464
SG009	3571	SG055	1834	SG101	6	SG147	1003	SG193	1682
SG010	353	SG056	1157	SG102	55	SG148	5509	SG194	10463
SG011	4046	SG057	949	SG103	18	SG149	52	SG195	43
SG012	10698	SG058	1274	SG104	59	SG150	539	SG196	69
SG013	3347	SG059	902	SG105	8	SG151	88	SG197	453
SG014	109650	SG060	3442	SG106	48	SG152	491	SG198	1248
SG015	34041	SG061	9236	SG107	418	SG153	9	SG199	163
SG016	27	SG062	19063	SG108	82	SG154	282	SG200	188
SG017	71	SG063	5498	SG109	146	SG155	598	SG201	255
SG018	1303	SG064	1575	SG110	169	SG156	186	SG202	139
SG019	14363	SG065	1194	SG111	36	SG157	107	SG203	324
SG020	811	SG066	466	SG112	62	SG158	74	SG204	1437
SG021	82	SG067	150	SG113	633	SG159	424	SG205	1223
SG022	52	SG068	229	SG114	473	SG160	1506	SG206	837
SG023	128	SG069	485	SG115	127	SG161	1523	SG207	8
SG024	37	SG070	986	SG116	39	SG162	278	SG208	23
SG025	72	SG071	2015	SG117	67	SG163	507	SG209	32
SG026	273	SG072	695	SG118	116	SG164	2589	SG210	36
SG027	13	SG073	174	SG119	186	SG165	646	SG211	29
SG028	41	SG074	1480	SG120	70	SG166	8110	SG212	161
SG029	2212	SG075	62	SG121	489	SG167	4357	SG213	407
SG030	55	SG076	242	SG122	1017	SG168	29	SG214	149
SG031	675	SG077	31	SG123	4273	SG169	206	SG215	334
SG032	131	SG078	199	SG124	87	SG170	169	SG216	4217
SG033	4703	SG079	130	SG125	227	SG171	25	SG217	1037
SG034	223	SG080	114	SG126	218	SG172	17	SG218	532
SG035	68	SG081	113	SG127	1945	SG173	1765	SG219	113
SG036	1496	SG082	1076	SG128	422	SG174	572	SG220	1480
SG037	105	SG083	121	SG129	3376	SG175	192	SG221	7850
SG038	447	SG084	105	SG130	425	SG176	3951	SG222	189
SG039	75	SG085	527	SG131	141	SG177	14	SG223	1670
SG040	227	SG086	746	SG132	57	SG178	143	SG224	113
SG041	495	SG087	1686	SG133	46	SG179	118	SG225	17484
SG042	113	SG088	2473	SG134	62	SG180	310	SG226	622
SG043	1374	SG089	11	SG135	162	SG181	33	SG227	12886
SG044	293	SG090	69	SG136	2170	SG182	332	SG228	119
SG045	232	SG091	73	SG137	583	SG183	24	SG229	2053
SG046	339	SG092	781	SG138	124	SG184	20	SG230	1981

Table S2: Number of compounds in each space group (SG) in the merged dataset.

1 Descriptor Calculation

Elemental attributes were extracted from literature[18, 5] and include both experimental as well as theoretically calculated values. The properties considered include:

Electronegativity Rahm[26]

Orbital s, p, d, f-orbital valence, s, p, d, f unfilled valence electrons

Atomic Atomic number, atomic weight, Zunger pseudopotential radii, mendeleev number, lattice constant, atomic enthalpy, heat of fusion, heat of vaporization, melting point, ionization energy, Rahm atomic radii, Pykko covalent radii, L quantum number, work function, critical nuclear charge, heat of formation, specific heat, space group number, dipole polarizability, total valence electrons, total unfilled electrons, C_6 dispersion coefficient[9], Pettifor number[8], Glawe number[8], GSvolume_pa (maximum DFT-computed volume), GSBandgap (mean DFT bandgap), GSenergy_pa (DFT computed energy), GSestBCClatcnt, GSestFCClatcnt, GSmagmom

Descriptor abbreviations:

rad_#_orb radius of the s/p/d/f orbitals

N#Valence Number of s/p/d/f valence electrons

N#UnfValence Number of s/p/d/f unfilled valence electrons

eneg_rahm Rahm electronegativity

mendeleevnum Mendeleev number

zungerad Zunger radius

spheat Specific Heat

totunfilledelec total unfilled electrons

totvalelec total valence electrons

critnuccharge Critical nuclear charge

sgn space group number

ioneng Ionization energy

atmwt atomic weight

rahmrad Rahm atomic radius

period Period number

elaff electron affinity

lquant L quantum number

htevap heat of evaporation

bp boiling point

latconst lattice constant

cohesiveng cohesive energy

mp melting point

htfusion heat of fusion

workfn work function

eden electron density

enthalpyAtomization enthalpy of atomization

Table S3: High entropy alloys used in this study for external validation.

Compound	Space Group
Si0.3200Ni0.1700Co0.1600Cr0.1800Fe0.1700	SG062
Si0.3200Ni0.2000Co0.1900Cr0.1200Fe0.1700	SG062
Cu0.0500Si0.3400Ni0.1600Co0.1600Cr0.1300Fe0.1600	SG062
Si0.3560Ni0.1680Co0.1680Cr0.1390Fe0.1680	SG062
Si0.6700Ni0.0900Co0.1800Fe0.0600	SG225
Mo0.0660Nb0.0660Ti0.0660Si0.6700W0.0660Ta0.0660 [6]	SG181
Si0.7000Ni0.0200Co0.0300Cr0.0100Fe0.2400	SG123
Mo0.1670Nb0.1670V0.1670Zr0.1670N0.1670Cr0.1670 [25]	SG225
Ti0.1670Zr0.1670Al0.1670Ta0.1670N0.1670Cr0.1670 [25]	SG225
Ti0.1670V0.1670Si0.1670Al0.1670N0.1670Cr0.1670 [25]	SG225
Mo0.1670Ti0.1670Si0.1670Al0.1670N0.1670Cr0.1670 [25]	SG225
Nb0.1430Ti0.1430V0.1430Si0.1430Al0.1430N0.1430Cr0.1430 [25]	SG225
Ti0.1670V0.1670Zr0.1670Hf0.1670N0.1670Cr0.1670 [25]	SG225
Mo0.1430Ti0.1430Zr0.1430Al0.1430Ta0.1430N0.1430Cr0.1430 [25]	SG225
Ti0.1670V0.1670Zr0.1670Ta0.1670N0.1670Cr0.1670 [25]	SG225
Nb0.1670Ti0.1670V0.1670Zr0.1670Hf0.1670N0.1670 [25]	SG225
B0.1670Ti0.1670Si0.1670Al0.1670N0.1670Cr0.1670 [25]	SG225
Nb0.1670Ti0.1670Zr0.1670Ta0.1670Hf0.1670N0.1670 [25]	SG225
Nb0.1670Ti0.1670Si0.1670Al0.1670N0.1670Cr0.1670 [25]	SG225
Mo0.1670Ti0.1670Al0.1670Ta0.1670N0.1670Cr0.1670 [25]	SG225
C0.5000Nb0.1000Ti0.1000Zr0.1000Ta0.1000Hf0.1000 [25]	SG225
Mo0.1000C0.5000Nb0.1000V0.1000W0.1000Ta0.1000 [25]	SG225
C0.5000Ti0.1000W0.1000Zr0.1000Ta0.1000Hf0.1000 [25]	SG225
C0.5000Nb0.1250Zr0.1250Ta0.1250Hf0.1250 [25]	SG225
C0.5010Nb0.1660Zr0.1660Ta0.1660 [25]	SG225
C0.4550Nb0.0910Ti0.0910V0.0910Zr0.0910Ta0.0910Hf0.0910 [25]	SG225
C0.5000Nb0.1000Ti0.1000W0.1000Ta0.1000Cr0.1000 [25]	SG225
C0.5000Nb0.1000Ti0.1000Si0.1000Zr0.1000Cr0.1000 [25]	SG225
C0.5000Nb0.1000Ti0.1000Si0.1000Zr0.1000Ta0.1000 [25]	SG225
B0.6700Mo0.0660Ti0.0660Zr0.0660Ta0.0660Hf0.0660 [7]	SG191
B0.6700Mo0.0660Nb0.0660Ti0.0660Zr0.0660Hf0.0660 [7]	SG191
B0.6700Mo0.0660Nb0.0660Ti0.0660Ta0.0660Hf0.0660 [7]	SG191
B0.6700Mo0.0660Nb0.0660Ti0.0660Zr0.0660Ta0.0660 [7]	SG191
B0.6700Ti0.0660Zr0.0660Ta0.0660Hf0.0660Cr0.0660 [7]	SG191
Cu0.0060Al0.6450Ni0.0060Co0.0060O0.3230Cr0.0060Fe0.0060 [25]	SG206
Cu0.1110Ni0.1110Co0.1110O0.5550Cr0.1110Fe0.0010 [21]	SG225
Ce0.0800Pr0.0800La0.0800Y0.0800Sm0.0800O0.6000 [1]	SG225
Ni0.1000Co0.1000Ge0.1000O0.5000Cr0.1000Fe0.1000 [22]	SG225
Cu0.1000Zn0.1000Mg0.1000Ni0.1000Co0.1000O0.5000 [27]	SG225

Continued on next page

Table S3 – *Continued from previous page*

Compound	Space Group
Nb0.0400Ti0.0400Zr0.0400Sn0.0400Hf0.0400O0.6000Ba0.2000 [28]	SG225
Dy0.0800Ho0.0800Gd0.0800Er0.0800Tb0.0800O0.6000 [25]	SG206
Mn0.0850Zn0.0850Co0.0850O0.5730Cr0.0850Fe0.0850 [25]	SG227
Zn0.0850Ni0.0850Co0.0850O0.5730Cr0.0850Fe0.0850 [25]	SG227
Mn0.0850Ni0.0850Co0.0850O0.5730Cr0.0850Fe0.0850 [25]	SG227
Mn0.0400Ni0.0400Co0.0400Gd0.2000O0.6000Cr0.0400Fe0.0400 [31]	SG062
Mn0.0360La0.2730Ni0.0360Co0.0360O0.5450Cr0.0360Fe0.0360 [31]	SG062
Mn0.0400Nd0.2000Ni0.0400Co0.0400O0.6000Cr0.0400Fe0.0400 [31]	SG062
Se0.5000Ag0.1670Bi0.1670Ge0.1670 [25]	SG225
Mn0.1310Zn0.2020Ni0.1310Co0.0910Sb0.3330Fe0.1110	SG225
Ni0.1700Co0.1900Sb0.4600Fe0.1800	SG194
Ru0.1880Re0.2080Os0.2180Rh0.1980Ir0.1880 [34]	SG194
Ru0.1050Pt0.3260Os0.0530Rh0.2420Ir0.2740 [34]	SG225
Cu0.1100Ni0.1100Co0.1100O0.5490Cr0.1100Fe0.0110 [21]	SG225
Mn0.0420La0.1670Ni0.7920 [35]	SG191
Gd0.0220Ba0.0310O0.5940Fe0.3530 [19]	SG194
Mn0.2000Pr0.0200La0.1100Sr0.0700O0.6000 [10]	SG167
Mn0.1900Pr0.0200Ti0.0100La0.1100Sr0.0700O0.6000 [10]	SG167
C0.0830Nb0.3330W0.1670Ta0.3330Hf0.0830 [32]	SG225
Re0.5600Nb0.1100Ti0.1100Zr0.1100Hf0.1100 [20]	SG194
Nb0.0500Ti0.3500Zr0.2750Ta0.0500Hf0.2750 [14]	SG063
B0.3300Mo0.1100Re0.3400V0.1100W0.1100 [23]	SG140
Nb0.2000Ti0.2000Zr0.2000Ta0.2000Hf0.2000 [36]	SG229
Mo0.3000Ru0.2000Re0.3500Ta0.1000Cr0.0500 [16]	SG136
Mn0.2000Si0.0800Ni0.3300Ge0.2500Cr0.1400 [29]	SG062
Mn0.2400Si0.0800Ni0.3300Ge0.2500Cr0.1000 [29]	SG062
Nb0.2500Ti0.2500V0.2500Zr0.2500 [24]	SG229
Nb0.3330Ti0.3330V0.3330 [24]	SG229
Nb0.2000Ti0.2000V0.2000Zr0.2000Hf0.2000 [24]	SG229
Mo0.3130Ru0.3980Pd0.1000Nb0.1180Rh0.0710 [15]	SG194
Mo0.3230Ru0.3470Pd0.1000Nb0.1390Rh0.0910 [15]	SG194
Mo0.2820Ru0.3360Pd0.1030Nb0.2030Rh0.0760 [15]	SG194
Al0.1670Ni0.3330Co0.1670Cr0.1670Fe0.1670 [11]	SG221
Mn0.1670Al0.1670Ni0.1670Co0.1670Cr0.1670Fe0.1670 [30]	SG225
Nb0.0300Ti0.3030V0.0300Zr0.3030Ta0.0300Hf0.3030 [13]	SG194
Ti0.3000V0.3500Cr0.2500Fe0.1000 [17]	SG194
Mn0.1000Ti0.3000V0.3500Cr0.2500 [17]	SG194
Nb0.0500Ti0.3000V0.3000Cr0.2500Fe0.1000 [17]	SG194
Mn0.0500Ti0.3000V0.3500Cr0.2500Fe0.0500 [17]	SG194
Nb0.1670Ti0.3330V0.1670Zr0.3330 [2]	SG229

Continued on next page

Table S3 – *Continued from previous page*

Compound	Space Group
Mn0.2000Ni0.2000Co0.2000Cr0.2000Fe0.2000 [12]	SG136
Mo0.1500Ru0.1500Re0.3500Nb0.2500Rh0.1000 [17]	SG217
Mo0.1900Ru0.3330Re0.3330Nb0.0480Rh0.0950 [17]	SG194
Mo0.1290C0.0650Nb0.3230W0.1610Ta0.3230 [33]	SG194
Ce0.2000Ru0.2000Pt0.2000Pd0.2000Rh0.2000 [4, 3]	SG225
Cu0.1670Au0.1670Pt0.1670Ni0.1670Co0.1670Fe0.1670 [4, 3]	SG225
Cu0.1670Au0.1670Pt0.1670Pd0.1670Ni0.1670Co0.1670 [4, 3]	SG225
Cu0.1250Au0.1250Pt0.1250Pd0.1250Sn0.1250Ni0.1250Co0.1250Fe0.1250 [4, 3]	SG225
Pt0.2000La0.2000Ni0.2000Co0.2000Fe0.2000 [4, 3]	SG225
Ru0.2000Au0.2000Pt0.2000Pd0.2000Rh0.2000 [4, 3]	SG225
Ru0.2000Pt0.2000Pd0.2000Rh0.2000Ir0.2000 [4, 3]	SG225
Cu0.2000Mo0.2000Au0.2000Pd0.2000Al0.2000 [4, 3]	SG225
Pt0.2000Pd0.2000Ir0.2000Co0.2000Fe0.2000 [4, 3]	SG225
Mo0.1000Ni0.2000Co0.3500Cr0.1500Fe0.2000 [4, 3]	SG225
Mo0.4000Ir0.2000Al0.2000Co0.2000 [4, 3]	SG225
Mn0.1670Nb0.1670Ni0.1670Co0.1670Cr0.1670Fe0.1670 [4, 3]	SG225
Mn0.1670Mo0.1670Ni0.1670Co0.1670Cr0.1670Fe0.1670 [4, 3]	SG225
Cu0.1670Au0.1670Pt0.1670Pd0.1670Al0.1670Ni0.1670 [4, 3]	SG225
Cu0.2000Au0.2000Pt0.2000Pd0.2000Ag0.2000 [4, 3]	SG225
Ru0.1670Pt0.1670Pd0.1670Os0.1670Rh0.1670Ir0.1670 [4, 3]	SG225
Mn0.2000Mg0.2000Ni0.2000Co0.2000Fe0.2000 [4, 3]	SG225
C0.1670Nb0.1670Ti0.1670Zr0.1670Ta0.1670Hf0.1670 [4, 3]	SG225
Nb0.1200Ti0.2050V0.0700Al0.0850N0.5000Cr0.0200 [4, 3]	SG225
Nb0.0850Ti0.1700V0.0600Al0.1550N0.5000Cr0.0300 [4, 3]	SG225
Mo0.2000Nb0.2000Ti0.2000Zr0.2000Hf0.2000 [4, 3]	SG221
Ni0.3330Co0.3330Fe0.3330 [4, 3]	SG225
Ni0.2500Co0.2500Cr0.2500Fe0.2500 [4, 3]	SG225
Mn0.2500Ni0.2500Co0.2500Fe0.2500 [4, 3]	SG225
Al0.0590Ni0.2350Co0.2350Cr0.2350Fe0.2350 [4, 3]	SG225
Al0.0770Ni0.3080Co0.3080Fe0.3080 [4, 3]	SG225
Mn0.0710Al0.0710Ni0.2860Co0.2860Fe0.2860 [4, 3]	SG225
Ti0.2000Ni0.2000Co0.2000Cr0.2000Fe0.2000 [4, 3]	SG225
Cu0.2000Ni0.2000Co0.2000Cr0.2000Fe0.2000 [4, 3]	SG225
Si0.0770Ni0.3080Co0.3080Fe0.3080 [4, 3]	SG225
Si0.0310Al0.0310Ni0.3130Co0.3130Fe0.3130 [4, 3]	SG225
Si0.0590Al0.0590Ni0.2940Co0.2940Fe0.2940 [4, 3]	SG225
Pd0.2000Ni0.2000Co0.2000Cr0.2000Fe0.2000 [4, 3]	SG225
Pd0.3330Ni0.1670Co0.1670Cr0.1670Fe0.1670 [4, 3]	SG225
Cu0.1820Ti0.0910Ni0.1820Co0.1820Cr0.1820Fe0.1820 [4, 3]	SG225
Cu0.0590Al0.0590Ni0.2940Co0.2940Fe0.2940 [4, 3]	SG225

Continued on next page

Table S3 – *Continued from previous page*

**Compound**	**Space Group**
Cu0.1050Al0.1050Ni0.2630Co0.2630Fe0.2630 [4, 3]	SG225
Cu0.1430Al0.1430Ni0.2380Co0.2380Fe0.2380 [4, 3]	SG225
Al0.2000Ni0.2000Co0.2000Cr0.2000Fe0.2000 [4, 3]	SG221
Al0.2380Ni0.1900Co0.1900Cr0.1900Fe0.1900 [4, 3]	SG221
Al0.3330Ni0.1670Co0.1670Cr0.1670Fe0.1670 [4, 3]	SG221
Nb0.0200Al0.1960Ni0.1960Co0.1960Cr0.1960Fe0.1960 [4, 3]	SG221

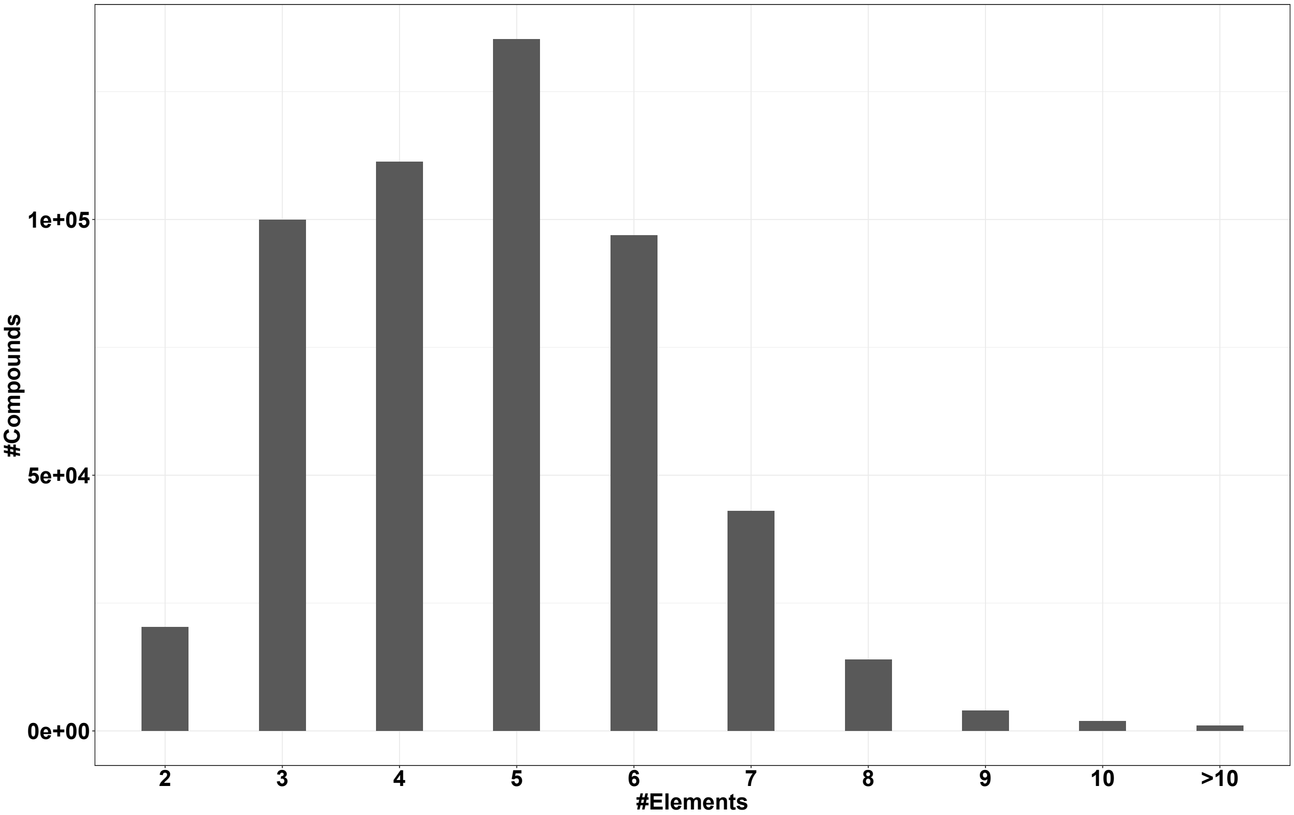

Figure F2: Bar plot showing the distribution of the compounds in the merged dataset with respect to the number of elements present.

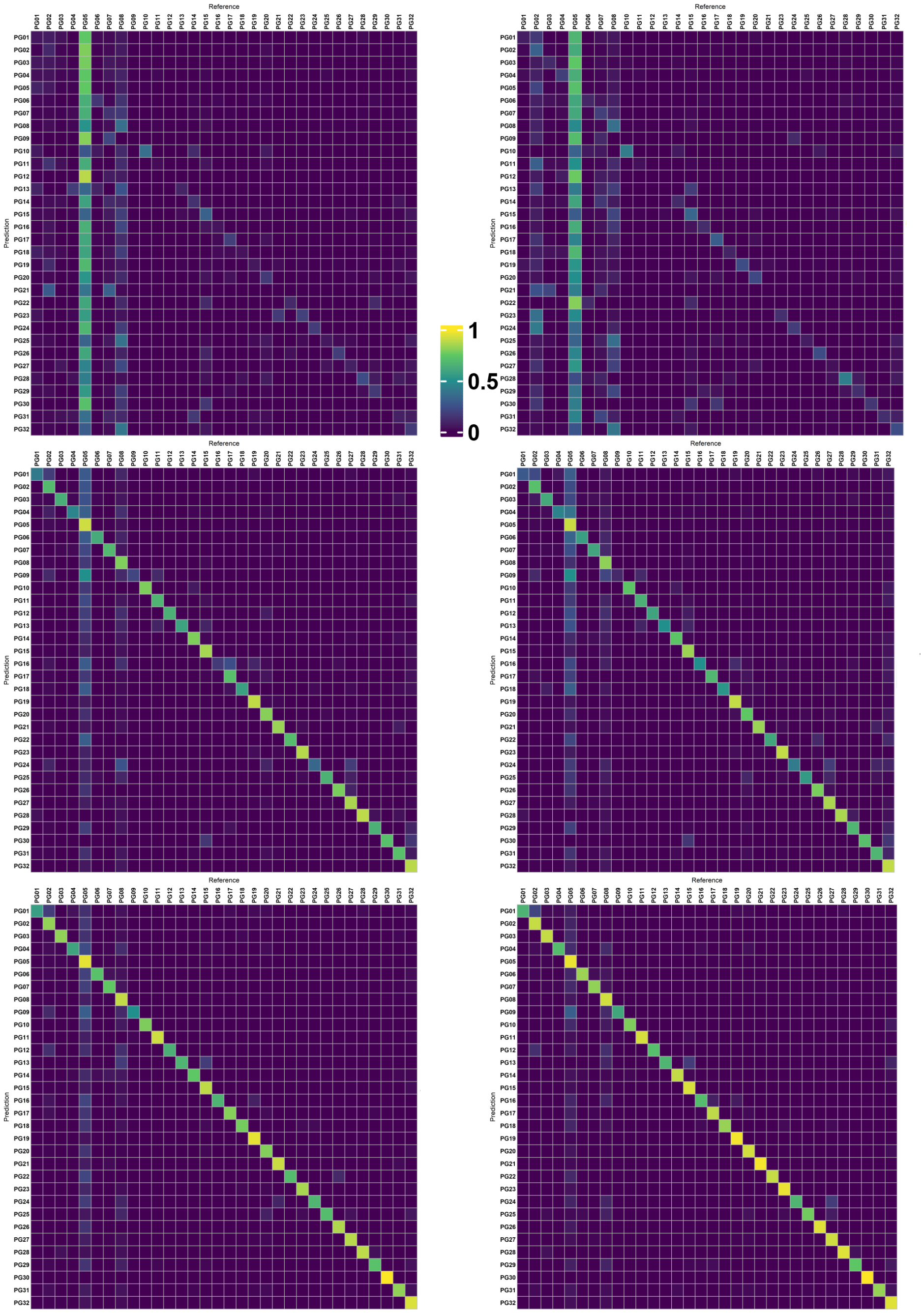

Figure F3: Confusion matrices corresponding to point group predictions provided by models trained on MP, OQMD, ICSD, PEARSON, COD and MERGED (left to right). Model predictions for the 32 point groups were made for over 8000 compounds (not present in the training data) from the American mineralogist crystal structure database.

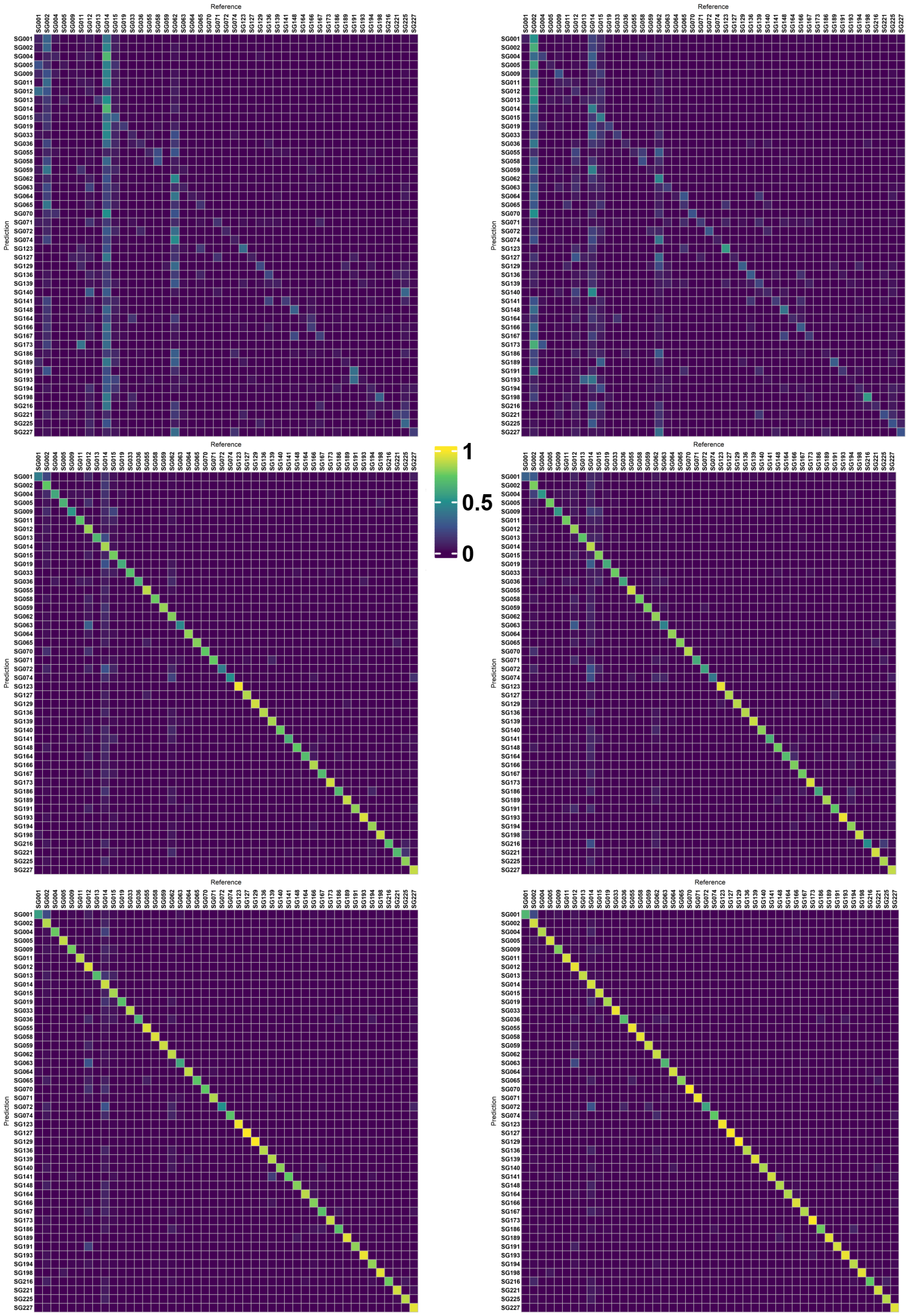

Figure F4: Confusion matrices corresponding to space group predictions provided by models trained on MP, OQMD, ICSD, PEARSON, COD and MERGED (left to right). Model predictions for the top 46 most frequently occurring space groups were made for over 8000 compounds (not present in the training data) from the American mineralogist crystal structure database.

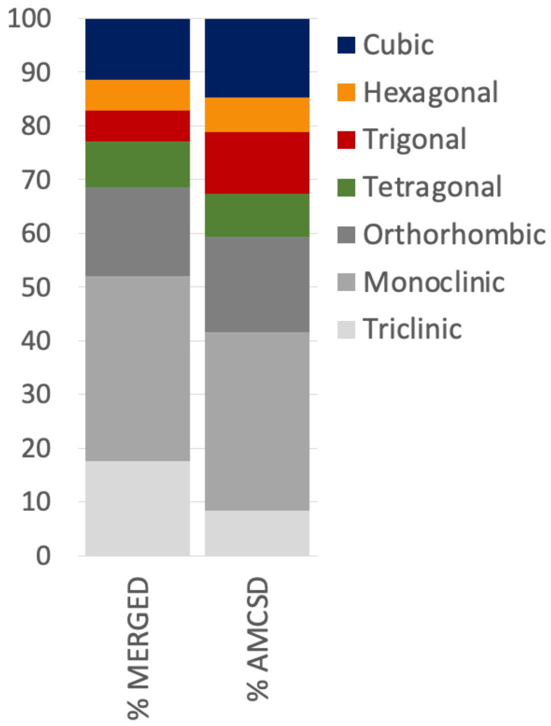

Figure F5: Distribution of compounds across crystal systems for MERGED vs AMSCD

Response	Top-1	Top-2	Top-3	Top-5
Bravais Lattice	0.597±0.0010	0.789±0.0006	0.883±0.0015	0.960±0.0006
Space group	0.557±0.0021	0.736±0.0020	0.808±0.0015	0.865±0.0015
Crystal System	0.661±0.0010	0.867±0.0012	0.956±0.0006	0.989±0.0006
Lattice centering	0.854±0.0012	0.964±0.0006	0.985±0.0006	–
Point group	0.617±0.0012	0.815±0.0010	0.875±0.0006	0.932±0.0001

Table S4: Top-k accuracies and standard deviations obtained for the 3 test splits. Results shown for the random forests model.

References

[1] M. R. Chellali, A. Sarkar, S. H. Nandam, S. S. Bhattacharya, B. Breitung, H. Hahn, and L. Velasco. On the homogeneity of high entropy oxides: An investigation at the atomic scale. *Scripta Materialia*, 166:58–63, 2019.

[2] Y. Chen, Z. Xu, M. Wang, Y. Li, C. Wu, and Y. Yang. A single-phase v0.5nb0.5zrti refractory high-entropy alloy with outstanding tensile properties. *Materials Science and Engineering: A*, 792:139774, 2020.

[3] M. Fu, X. Ma, K. Zhao, X. Li, and D. Su. High-entropy materials for energy-related applications. *iScience*, 24(3):102177, 2021.

[4] M. C. Gao, D. B. Miracle, D. Maurice, X. Yan, Y. Zhang, and J. A. Hawk. High-entropy functional materials. *Journal of Materials Research*, 33(19):3138–3155, 2018.

[5] E. Generalic. Periodic table of the elements, calculators, and printable materials. `http://www.periodni.com`, 2020. Online; accessed: 01.06.2020.

[6] J. Gild, J. Braun, K. Kaufmann, E. Marin, T. Harrington, P. Hopkins, K. Vecchio, and J. Luo. A high-entropy silicide: (mo0.2nb0.2ta0.2ti0.2w0.2)si2. *Journal of Materiomics*, 5(3):337–343, 2019.

[7] J. Gild, Y. Zhang, T. Harrington, S. Jiang, T. Hu, M. C. Quinn, W. M. Mellor, N. Zhou, K. Vecchio, and J. Luo. High-entropy metal diborides: A new class of high-entropy materials and a new type of ultrahigh temperature ceramics. *Scientific Reports*, 6(1), 2016.

[8] H. Glawe, A. Sanna, E. K. U. Gross, and M. A. L. Marques. The optimal one dimensional periodic table: a modified pettifor chemical scale from data mining. *New Journal of Physics*, 18(9):093011, 2016.

[9] T. Gould and T. Bučko. C6 coefficients and dipole polarizabilities for all atoms and many ions in rows 1–6 of the periodic table. *Journal of Chemical Theory and Computation*, 12(8):3603–3613, 2016.

[10] A. Guedri, S. Mnefgui, S. Hcini, E. Hlil, and A. Dhahri. B-site substitution impact on structural and magnetocaloric behavior of la0.55pr0.1sr0.35mn1-xTixO3 manganites. *Journal of Solid State Chemistry*, 297:122046, 2021.

[11] M. S. Jadhav, D. Sahane, A. Verma, and S. Singh. Thermal stability and thermal expansion behavior of FeCoCrNi2al high entropy alloy. *Advanced Powder Technology*, 32(2):378–384, 2021.

[12] J. Joseph, N. Haghdadi, M. Annasamy, S. Kada, P. Hodgson, M. Barnett, and D. Fabijanic. On the enhanced wear resistance of CoCrFeMnNi high entropy alloy at intermediate temperature. *Scripta Materialia*, 186:230–235, 2020.

[13] Y. Jung, K. Lee, S. J. Hong, J. K. Lee, J. Han, K. B. Kim, P. K. Liaw, C. Lee, and G. Song. Investigation of phase-transformation path in TiZrHf(VNbTa)x refractory high-entropy alloys and its effect on mechanical property. *Journal of Alloys and Compounds*, 886:161187, 2021.

[14] L. Lilensten, J. Couzinié, L. Perrière, J. Bourgon, N. Emery, and I. Guillot. New structure in refractory high-entropy alloys. *Materials Letters*, 132:123–125, 2014.

[15] B. Liu, J. Wu, Y. Cui, Q. Zhu, G. Xiao, S. Wu, G. Cao, and Z. Ren. Superconductivity in hexagonal nb-mo-ru-rh-pd high-entropy alloys. *Scripta Materialia*, 182:109–113, June 2020.

[16] B. Liu, J. Wu, Y. Cui, Q. Zhu, G. Xiao, S. Wu, G. han Cao, and Z. Ren. Superconductivity and paramagnetism in cr-containing tetragonal high-entropy alloys. *Journal of Alloys and Compounds*, 869:159293, 2021.

[17] J. Liu, J. Xu, S. Sleiman, X. Chen, S. Zhu, H. Cheng, and J. Huot. Microstructure and hydrogen storage properties of ti–v–cr based BCC-type high entropy alloys. *International Journal of Hydrogen Energy*, 46(56):28709–28718, 2021.

[18] K. LLC. www.knowledgedoor.com. `http://www.knowledgedoor.com`, 2020. Online; accessed 01.06.2020.

[19] M. K. Manglam and M. Kar. Effect of gd doping on magnetic and MCE properties of m-type barium hexaferrite. *Journal of Alloys and Compounds*, 899:163367, 2022.

[20] S. Marik, K. Motla, M. Varghese, K. P. Sajilesh, D. Singh, Y. Breard, P. Boullay, and R. P. Singh. Superconductivity in a new hexagonal high-entropy alloy. *Physical Review Materials*, 3(6), 2019.

[21] J. Mayandi, M. Dias, M. Stange, A. Lind, M. F. Sunding, A. Cerdeira, M. Schrade, B. Belle, T. Finstad, L. Pereira, S. Diplas, and P. A. Carvalho. Partial oxidation of high entropy alloys. a route towards nanostructured ferromagnets? *Materilia*, page 101250, 2021.

[22] J. Mayandi, T. Finstad, Ø. Dahl, P. Vajeeston, M. Schrade, O. Løvvik, S. Diplas, and P. Carvalho. Thin films made by reactive sputtering of high entropy alloy FeCoNiCuGe: Optical, electrical and structural properties. *Thin Solid Films*, 744:139083, 2022.

[23] K. Motla, V. Soni, P. K. Meena, and R. P. Singh. Boron based new high entropy alloy superconductor mo0.11w0.11v0.11re0.34b0.33. *Superconductor Science and Technology*, 35(7):074002, 2022.

[24] M. M. Nygård, W. A. Sławiński, G. Ek, M. H. Sørby, M. Sahlberg, D. A. Keen, and B. C. Hauback. Local order in high-entropy alloys and associated deuterides – a total scattering and reverse monte carlo study. *Acta Materialia*, 199:504–513, 2020.

[25] C. Oses, C. Toher, and S. Curtarolo. High-entropy ceramics. *Nature Reviews Materials*, 5(4):295–309, 2020.

[26] M. Rahm, T. Zeng, and R. Hoffmann. Electronegativity seen as the ground-state average valence electron binding energy. *Journal of the American Chemical Society*, 141(1):342–351, 2018.

[27] C. M. Rost, E. Sachet, T. Borman, A. Moballegh, E. C. Dickey, D. Hou, J. L. Jones, S. Curtarolo, and J.-P. Maria. Entropy-stabilized oxides. *Nature Communications*, 6(1), 2015.

[28] Y. Sharma, B. L. Musico, X. Gao, C. Hua, A. F. May, A. Herklotz, A. Rastogi, D. Mandrus, J. Yan, H. N. Lee, M. F. Chisholm, V. Keppens, and T. Z. Ward. Single-crystal high entropy perovskite oxide epitaxial films. *Physical Review Materials*, 2(6), 2018.

[29] A. Tekgül, K. Sarlar, N. Küçük, and A. B. Etemoğlu. The structural, magnetic and magnetocaloric properties of MnCrNiGeSi high-entropy alloy. *Physica Scripta*, 97(7):075814, 2022.

[30] S. Uporov, R. Ryltsev, V. Bykov, S. K. Estemirova, and D. Zamyatin. Microstructure, phase formation and physical properties of AlCoCrFeNiMn high-entropy alloy. *Journal of Alloys and Compounds*, 820:153228, 2020.

[31] R. Witte, A. Sarkar, R. Kruk, B. Eggert, R. A. Brand, H. Wende, and H. Hahn. High-entropy oxides: An emerging prospect for magnetic rare-earth transition metal perovskites. *Physical Review Materials*, 3(3), 2019.

[32] S. Wu, D. Qiao, H. Zhang, J. Miao, H. Zhao, J. Wang, Y. Lu, T. Wang, and T. Li. Microstructure and mechanical properties of c hf0.25nbtaw0.5 refractory high-entropy alloys at room and high temperatures. *Journal of Materials Science & Technology*, 97:229–238, 2022.

[33] S. Wu, D. Qiao, H. Zhao, J. Wang, and Y. Lu. A novel NbTaW0.5 (mo2c)x refractory high-entropy alloy with excellent mechanical properties. *Journal of Alloys and Compounds*, 889:161800, 2021.

[34] K. V. Yusenko, S. Riva, P. A. Carvalho, M. V. Yusenko, S. Arnaboldi, A. S. Sukhikh, M. Hanfland, and S. A. Gromilov. First hexagonal close packed high-entropy alloy with outstanding stability under extreme conditions and electrocatalytic activity for methanol oxidation. *Scripta Materialia*, 138:22–27, 2017.

[35] S. Zhu, X. Chen, J. Liu, N. Yang, J. Chen, C. Gu, H. Cheng, K. Yan, Z. Zhu, and K. Wang. Long-term hydrogen absorption/desorption properties of an AB5-type LaNi4.75mn0.25 alloy. *Materials Science and Engineering: B*, 262:114777, 2020.

[36] C. Zlotea, M. Sow, G. Ek, J.-P. Couzinié, L. Perrière, I. Guillot, J. Bourgon, K. Møller, T. Jensen, E. Akiba, and M. Sahlberg. Hydrogen sorption in TiZrNbHfTa high entropy alloy. *Journal of Alloys and Compounds*, 775:667–674, 2019.
